# Integrated qPCR and Staining Methods for Detection and Quantification of *Enterocytozoon hepatopenaei* in Shrimp *Litopenaeus vannamei*

**DOI:** 10.3390/microorganisms8091366

**Published:** 2020-09-07

**Authors:** Lijun Wang, Qing Lv, Yantong He, Ruocheng Gu, Bingqian Zhou, Jie Chen, Xiaodong Fan, Guoqing Pan, Mengxian Long, Zeyang Zhou

**Affiliations:** 1 State Key Laboratory of Silkworm Genome Biology, Southwest University, Chongqing 400715, China; wanglijunmail96@163.com (L.W.); lvaqing2020@163.com (Q.L.); honeababy@icloud.com (Y.H.); guruocheng96@163.com (R.G.); 15139498130@163.com (B.Z.); jchen@swu.edu.cn (J.C.); gqpan@swu.edu.cn (G.P.); zyzhou@swu.edu.cn (Z.Z.); 2 Chongqing Key Laboratory of Microsporidia Infection and Control, Southwest University, Chongqing 400715, China; fanxd@cqnu.edu.cn; 3 College of Life Sciences, Chongqing Normal University, Chongqing 401331, China

**Keywords:** *Enterocytozoon hepatopenaei*, gastrointestinal pathogen, fluorescence quantitative PCR, polar tube protein 2, fluorescent brightener

## Abstract

*Enterocytozoon hepatopenaei* (EHP) is an obligate, intracellular, spore-forming parasite, which mainly infects the gastrointestinal tract of shrimp. It significantly hinders the growth of shrimp, which causes substantial economic losses in farming. In this study, we established and optimized a SYBR Green I fluorescent quantitative PCR (qPCR) assay based on the *polar tube protein 2* (*PTP2*) gene for the quantitative analysis of EHP-infected shrimp. The result showed that the optimum annealing temperature was 60 °C for the corresponding relation between the amplification quantitative (*Cq*) and the logarithmic of the initial template quantity (*x*), conformed to *Cq* = −3.2751*x* + 31.269 with a correlation coefficient *R*^2^ = 0.993. The amplification efficiency was 102%. This qPCR method also showed high sensitivity, specificity, and repeatability. Moreover, a microscopy method was developed to observe and count EHP spores in hepatopancreas tissue of EHP-infected shrimp using Fluorescent Brightener 28 staining. By comparing the PTP2-qPCR and microscopy method, the microscopic examination was easier to operate whereas PTP2-qPCR was more sensitive for analysis. And we found that there was a correspondence between the results of these two methods. In summary, the PTP2-qPCR method integrated microscopy could serve for EHP detection during the whole period of shrimp farming and satisfy different requirements for detecting EHP in shrimp farming.

## 1. Introduction

Microsporidia are obligate, intracellular, spore-forming parasites, and diverse species infect almost all invertebrates and vertebrates, as well as some protists, with different species exhibiting various degrees of host specificity [1,2]. It is currently considered as a kind of fungi, and approximately 200 genera and 1400 species have been identified [3,4,5]. *Enterocytozoon hepatopenaei* (EHP), first discovered in Thailand *Penaeus monodon* [6,7], mainly infects the gastrointestinal tract of shrimp [8]. Although EHP is not a fatal pathogen for shrimp, in fact, it can spread horizontally in shrimp ponds by cannibalism and cohabitation, seriously affecting the development of shrimp, which may bring substantial economic losses for shrimp farmers [9]. Nowadays, EHP has been also reported in some other countries such as China, Vietnam, Brunei, Malaysia, Indonesia, India, and Venezuela [10,11,12,13].

EHP is closely related to *Ent. bieneusi,* which is known to infect immune-suppressed and immunodeficient humans, such as patients with AIDS [14]. Most microsporidian infections in humans are zoonotic and/or water-borne [4]. Although there is no evidence showing that EHP infects other animals except shrimp, for humans’ health, it is extremely important to detect EHP in shrimp. Due to the absence of obvious clinical symptoms in a short time frame, healthy shrimp may be infected with EHP by cohabiting with diseased shrimp [15,16]. Therefore, it is necessary to develop an efficient method to detect EHP-infected shrimp, especially for the early stage of infection. Currently, EHP detection methods have been reported via microscopy and molecular diagnosis. EHP spores could be stained and visualized by Phloxin B and calcofluor white (CFW) [17,18]. However, the microscopic examination mainly depends on the professional skill and subjective judgment of technicians. The sensitivity and specificity of microscopy are too limited and may misjudge the result. Furthermore, the higher sensitivity and specificity of molecular diagnoses have been widely reported for EHP-infected shrimp detection, such as PCR, qPCR, nested PCR, loop-mediated isothermal amplification (LAMP), and so on [19,20,21]. The small subunit ribosomal RNA (*SSU rRNA*) gene, a housekeeping gene, is a universal diagnostic target in EHP molecular detection methods. But it is well known that the *SSU rRNA* gene is highly conserved among microsporidia, which may give false-positive test results [22]. Hence, instead of *SSU rRNA*, a more specific diagnosis target needs to be chosen.

The polar tube, a highly specialized invasion organ, is one of the important taxonomic indexes of microsporidia [23]. Up to now, there are five polar tube proteins (PTP1–PTP5) located on the polar tube identified [4,24,25,26]. *PTP2* gene encoding a 35-kDa protein was first identified from microsporidium *Encephalitozoon cuniculi* [24]. This gene was also found in some other microsporidian genomes, involving *Enc. intestinalis*, *Enc. hellem*, *Paranosema grylli*, *Nosema ceranae*, *N. bombycis,* and so on [27,28,29]. The *PTP2* gene was also reported to be a single copy in the EHP genome [30]. Due to these unique properties, the *PTP2* gene was selected as the EHP detection target for recombinase polymerase amplification (RPA) and CRISPR-Cas 12a fluorescence assay [30]. However, this newly developed method cannot quantify the spore numbers of EHP in shrimp. In order to provide a more sensitive and specific EHP quantitative method, we established a SYBR Green I fluorescence quantitative PCR method based on the *PTP2* gene sequence in this study. Moreover, to provide real-time monitoring of EHP in the field, we attempted to quantify the EHP spores using microscopy. The integrated method of qPCR and microscopy to quantify EHP spores is first reported in our study and will provide a reference for the detection of EHP in shrimp farming.

## 2. Materials and Methods

### 2.1. Samples Treatment and Dna Extraction

We collected shrimp from Chongqing Province, China. Thirty mg of hepatopancreas tissue was used for genomic DNA extraction as follows: add 500 µL CTAB (CATB 4 g, NaCl 16.34 g, 1 M Tris-HCl (pH 8.0) 20 mL, 0.5 M EDTA 8 mL, sterilized water up to 200 mL) and 20 µL Proteinase K (20 mg/mL) before incubation at 56 °C for an hour. Total DNA was purified using a standard phenol-chloroform method [22].

### 2.2. Synthesis of Primers and Conventional PCR Amplification

The *EHP-PTP2* gene (GenBank No. MT249228), *SSU rRNA* gene (GenBank No. FJ496359.1) and *β-Tubulin* gene (GenBank No. KY593130) of EHP were amplified via PCR to confirm the EHP-infected shrimp sample. All PCR primers were designed using Primer Premier 5.0 and are listed in Table 1. The amplification system was 25 µL PrimeSTAR premix DNA polymerase (2×, TaKaRa, Dalian, China), 0.4 µM primers, 1 µL genomic DNA extraction, and water up to 50 µL. The amplification reaction was performed according to the following procedure: 98 °C for 5 min, 35 cycles (98 °C for 30 s, 56 °C for 30 s, 72 °C for 10 s), 72 °C for 10 min.

### 2.3. Construction of the Standard Sample

The amplified DNA fragment of the partial *EHP-PTP2* sequence (238 bp) was inserted into the pMD19-T vector and transformed into *Escherichia coli* DH5α. Positive colonies were selected to extract the plasmid and verified via sequencing (Sangon, Shanghai, China). The recombinant plasmid was extracted by the Mini Plasmid Extraction Kit (Omega, Norcross, GA, USA) and determined with a spectrophotometer (DeNovix, Wilmington, NC, USA) to be 54.6 ng/µL, which was equal to 1.7 × 10^10^ copies/µL. The recombinant plasmid was used as the quantitative standard and stored at −80 °C.

### 2.4. Optimization of the Reaction System

The reaction mixture of PTP2-qPCR was formulated on ice according to the description of Hieff^®^ qPCR SYBR Green Master Mix kit (Yeasen, Shanghai, China). The final concentration of EHP-PTP2-F and EHP-PTP2-R primers was 0.2 μM in the optimized reaction system (Table 2). The other components including 2 × Hieff^®^ qPCR SYBR Green Master Mix 5 μL, standard plasmid DNA template (1.0 × 10^3^ copies/μL) 1 μL, and nuclease-free water were added to make a total volume of 10 μL. The amplification reaction was performed in the LightCycler^®^ 96 (Roche, Indianapolis, IN, USA) and the optimized reaction procedure was 95 °C for 5 min, followed by 40 cycles of 95 °C for 10 s and 60 °C for 30 s. The data analysis was performed using the LightCycler^®^ 96 Software 1.1 (Roche).

### 2.5. Generation of the Standard Curve

The standard plasmid was diluted to 1.0 × 10^7^ copies/μL and made a 10-fold series of 7 gradients (1.0 × 10^7^–1.0 × 10^1^ copies/μL). Three parallels of each dilution were used as the template of the qPCR assays. A standard curve corresponding to the *Cq* value of the standard plasmid copy number was constructed. The correlation coefficient and amplification efficiency were also analyzed.

### 2.6. Specificity Analysis

To analyze the specificity of PTP2-qPCR, the total DNA of *L. vannamei* infected with different shrimp pathogens such as white spot syndrome virus (WSSV), shrimp hemocyte iridescent virus (SHIV), as well as *Vibrio parahaemolyticus* (VP_AHPND_) causing acute hepatopancreatic necrosis disease were used as templates to conduct qPCR amplification. The total DNA of healthy *L. vannamei* was used as the template of the negative control, the total DNA of EHP-infected *L. vannamei* was used as the template of the positive control, and the blank control used water as the template, respectively.

### 2.7. Sensitivity Analysis

In order to determine the sensitivity of PTP2-qPCR, serially diluted positive plasmids DNA (1.0 × 10^5^–1.0 × 10^1^ copies/μL) were used as qPCR templates, and water was used as the negative control. The highest dilution which could be detected while still showing an S-shaped amplification of the curves was considered the lowest template copy concentration of the qPCR. The same test was performed by conventional PCR, and the highest dilution that could provide a visible band on the agarose gel was equivalent to the lowest template copy concentration of the PCR.

### 2.8. Repeatability Analysis

To analyze the repeatability of the PTP2-qPCR, three different experimental personnel performed qPCR detection. Five EHP-infected *L. vannamei* were used as qPCR samples, and the standard deviation and coefficient of variation of the operator were calculated.

### 2.9. Microscopy Analysis

Regarding the microsporidian chitin-staining method [31], 0.8 mg of hepatopancreas tissue was ground and added on a 0.01% poly-lysine coated slide. Then, samples were covered with 50 µL solution of 4% paraformaldehyde and 50% Triton (49:1; *v/v*) at room temperature for 25 min, followed by washing with PBS (pH 7.0) three times. Fifty µL of Fluorescent Brightener 28 (1:1000 dilution; Sigma, St. Louis, MO, USA) was added and incubated for 5 min, then washed with PBS (pH 7.0) three times. EHP spores were observed by the fluorescent microscope (Olympus BX53F, Tokyo, Japan), and the spore number in twenty random fields was recorded.

## 3. Results

### 3.1. qPCR Standard Curve

**Figure 1:**
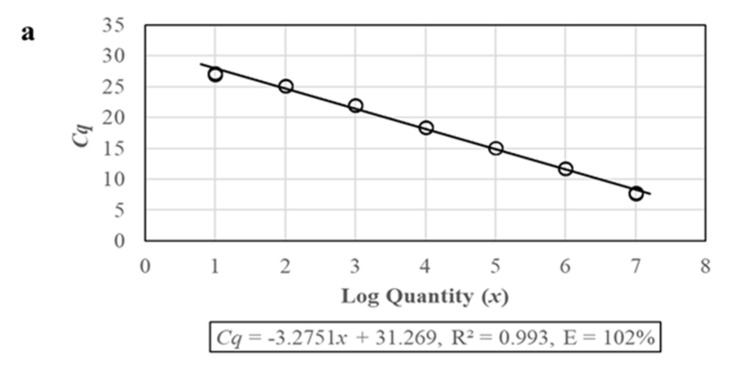

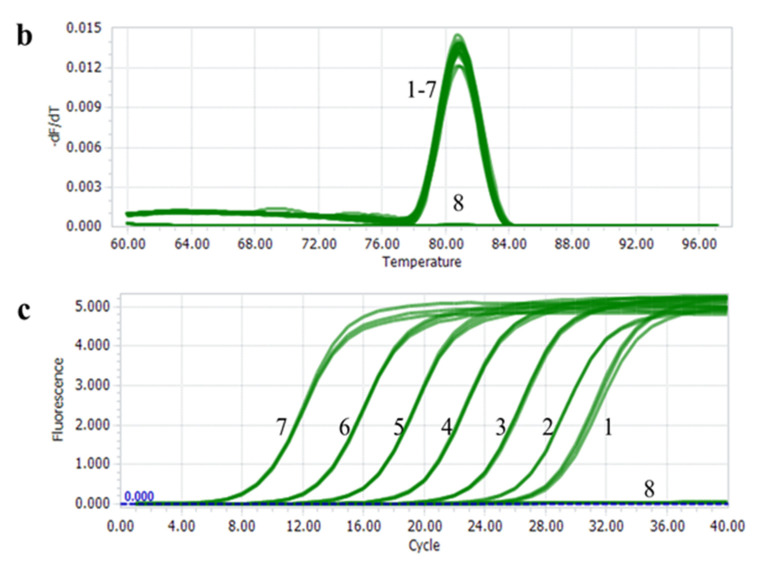
Amplification of the standard sample. (**a**) Standard curve of PTP2-qPCR. (**b**) Melting peaks of PTP2-qPCR. (**c**) Amplification curves of PTP2-qPCR. 1–7: 1.0 × 10^1^ to 1.0 × 10^7^ copies/μL standard plasmids. 8: water.

The optimized reaction system was used to establish the standard curve corresponding to the *Cq* value of the standard template copy number. The corresponding relation between the amplification quantitative (*Cq*) and the logarithmic of the initial template quantity (*x*) showed a good linear correlation when *x* was within the range of 1.0 × 10^1^ to 1.0 × 10^7^ copies/μL: *Cq* = −3.2751*x* + 31.269, correlation coefficient *R*^2^ = 0.993, and the amplification efficiency was 102%. From the amplification curve shown in Figure 1, there was a good gradient and a unique melting peak at 81 °C for the whole amplification process, indicating that the amplification products were uniform.

### 3.2. Specificity Analysis

**Figure 2:**
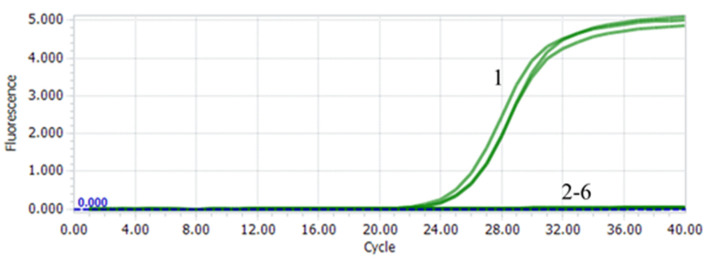
The specificity amplification curve of PTP2-qPCR. The templates for the qPCR were the DNA extracted from shrimp infected with 1. EHP; 2. WSSV; 3. SHIV; 4. VP_AHPND_; and 5. healthy shrimp; 6: water.

The specificity of the PTP2-qPCR method was analyzed using EHP and other shrimp pathogens including WSSV, SHIV, VP_AHPND_. Only the EHP positive template showed a significant amplification curve, while no fluorescent signal existed in other templates, indicating that this quantitative method had good specificity (Figure 2).

### 3.3. Sensitivity Analysis

**Figure 3:**
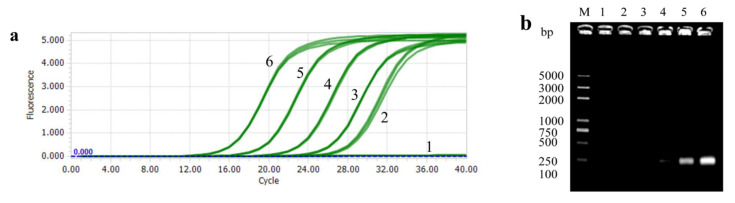
Sensitivity tests for the plasmid standard template. (**a**) The amplification curve of PTP2-qPCR. (**b**) Agarose electrophoresis of conventional PCR. 1: Negative control, 2–5: 1.0 × 10^1^ to 1.0 × 10^5^ copies/μL plasmid template.

With a typical S-shaped curve in the valid *Cq* range, the lowest template copy concentration detected by PTP2-qPCR was up to 1.0 × 10^1^ copies/μL (Figure 3a). With conventional PCR, it was difficult to distinguish the amplification fragment with the naked eye when the template was lower than 1.0 × 10^3^ copies/μL (Figure 3b). It was indicated that the sensitivity of PTP2-qPCR was at least two orders of magnitude higher than the conventional PCR.

### 3.4. Repeatability Analysis

From three different experimental personnel, the standard deviation (SD) and coefficient of variation (CV) were calculated by *Cq* values. The result showed that the *Cq* values of these three different experimental personnel were basically consistent; meanwhile, CV < 1%, suggesting that the repeatability of PTP2-qPCR was reliable (Table 3).

### 3.5. Integrated PTP2-qPCR and Microscopy Analysis EHP in Field-Shrimp

**Figure 4:**
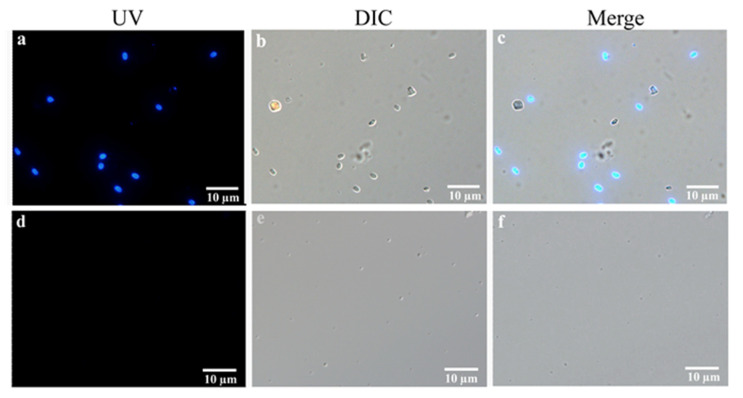
Staining analysis of EHP spores in the hepatopancreas of shrimp samples. (**a**–**c**) The hepatopancreas of EHP-infected shrimp samples staining with Fluorescent Brightener 28 on the UV light phase, differential interference contrast (DIC) phase, and a merged image. (**d**–**f**) The hepatopancreas of normal shrimp samples staining with Fluorescent Brightener 28 on the UV light phase, differential interference contrast (DIC) phase, and a merged image. Bar, 10 μm.

Stained by Fluorescent Brightener 28, many oval-shaped spores ranging in size from 1 to 2 μm were observed from EHP-infected shrimp, while there was no fluorescent signal in normal-shrimp samples (Figure 4). During analysis of the same EHP-infected sample via the integrated staining and PTP2-qPCR method, there was a simple correspondence between the spore number and the copy concentration of the *PTP2* gene (Table 4). It was difficult to observe EHP spores when the EHP concentration was lower than 10^3^ copies/mg. However, with the order of magnitude increase of the EHP concentration, the number of spores in one field also increased regularly. So, the EHP concentration would be quickly predicted according to the number of spores via microscopic examination when the shrimp were seriously infected.

## 4. Discussion

Microsporidia have been studied for more than 150 years. Various species can infect a wide variety of animals ranging from invertebrate to vertebrate [1,2,3,4,5,32]. EHP mainly parasitizes the hepatopancreas and gut of shrimp, causing the slowing growth of the host [6,7]. Since EHP does not cause rapid pathological changes in shrimp, it is difficult for farmers to quickly distinguish this pathogen [8]. For EHP detection, some microscopic examination methods were simply operated and broadly used [17,18]. However, microscopic examination with low sensitivity and accuracy was hard to detect EHP-infected shrimp, especially in the early infection. Therefore, high sensitivity molecular diagnoses such as PCR [33], qPCR [34], and loop-mediated isothermal amplification (LAMP) [35] have been developed to replace microscopy methods. *SSU rRNA* gene, a common target for EHP molecular diagnosis with a highly conserved sequence, was likely to produce false-positive results [19,34,36]. Hence, a specific target was selected in our study to establish a SYBR Green I fluorescence quantitative PCR method for EHP detection.

All microsporidia possess a unique, highly specialized structure: the polar tube. The polar tube is an important organ of the unique infection mechanism of microsporidia which can transport cytoplasm to host cells upon appropriate environmental stimulation [23]. Many kinds of polar tube proteins (PTPs) form the special structure, and these polar tube proteins play an important role in microsporidian invasion and proliferation [5]. EHP-PTP2 protein (GenBank No. OQS55341.1) had the highest identity (52%) with the homologous protein of other microsporidia by BlastP, implying the DNA sequence identity of their genes would be even lower. However, the *EHP-SSU rRNA* gene (GenBank No. KF362130.1) shared 93% identity with the *SSU rRNA* gene of *Enterospora nucleophile* (GenBank No. KF135641.1), and the identity shared with the other five microsporidia was higher than 85%. According to the latest report, the *PTP2* gene exhibited a good detection target in recombinase polymerase amplification (RPA) and CRISPR-Cas 12a fluorescence assay [30]. Actually, the EHP concentration is a key parameter in shrimp farming, and this latest approach cannot meet the requirements of quantitative detection. In this study, targeting of the *PTP2* gene of fluorescence SYBR Green I using real-time quantitative PCR was established to detect EHP, and the minimum copy concentration was up to 10 copies/μL EHP, which suggested this diagnosis had a high sensitivity. Additionally, there was no interference reaction with other shrimp pathogens verified in our study (Figure 2), implying this PTP2-qPCR approach had a good specificity.

One of our aims is to provide a more convenient EHP detection method for shrimp culture. Microscopy can be more accessible to detect pathogens in the field, as it does not require professional technicians and instruments. A field-portable and cost-effective smartphone-based platform was presented for the detection and quantification of chitin-positive *Nosema* spores in field measurements [37]. In this study, we used Fluorescent Brightener 28 to stain EHP spores in hepatopancreas tissue of EHP-infected shrimp and counted the EHP spores using microscopy. Combining the microscopy and PTP2-qPCR results, there were 40 to 50 spores in one field when 10^6^ copies/mg EHP could be detected by PTP2-qPCR, 7 to 20 spores vs. 10^5^ copies/mg, 2 to 6 spores vs. 10^4^ copies/mg, and 1 to 2 spores vs. 10^3^ copies/mg. When examining the number of spores via microscope, in this case, the EHP concentration would be easily predicted.

Above all, the use of the PTP2-qPCR method was recommended as early detection for EHP-infection, and staining microscopy was more suitable for real-time monitoring of EHP in the field. This integrated methodology could serve for EHP detection during the whole period of shrimp farming and provide a reference for the epidemiological study of EHP.

## 5. Conclusions

To our knowledge, this study is the first integrated qPCR and staining microscopy method for EHP detection. In shrimp culture, when EHP infection is serious, it can be directly detected by a microscope, and when EHP infection is mild, it can be detected by qPCR. The combination of these two methods not only makes the test results more accurate, but also prevents and controls EHP timely and effectively. We recommend that the integrated method be used to study EHP transmission routes in the shrimp–human food chain to monitor food chain safety.

## Figures and Tables

**Table 1:** The primer sequence in this study.

Primer	Sequence (5′-3′)	PCR Length
EHP-PTP2-F (qPCR)	GCAGCACTCAAGGAATGGC	238 bp
EHP-PTP2-R (qPCR)	TTTCGTTAGGCTTACCCTGTGA	
EHP-PTP2-F	ATGAGTCTTTATAATGCACTG	855 bp
EHP-PTP2-R	TTATTCGTTGGATGTTAATG	
EHP-SSU-F	GATGGCTCCCACGTCCAAGG	913 bp
EHP-SSU-R	GAACAGGGACACATTCACAA	
EHP-Tubulin-F	ATGAGAGAAATTATTCATGTACAGG	1317 bp
EHP-Tubulin-R	TTAATAACCTCCTTCTTCAATAAC	

**Table 2:** The reaction system of PTP2-qPCR (10 μL).

Reaction System	
2×Hieff^®^ qPCR SYBR Green Master Mix	5.0 μL
EHP-PTP2-F	0.2 μM
EHP-PTP2-R	0.2 μM
Template DNA	1.0 μL
ddH_2_O	Add to 10 μL

**Table 3:** The coefficient of variation (CV) analysis of PTP2-qPCR.

Sample No.	People	Mean *Cq* Value ± S	*Cq* SD	CV/%
1	3	29.64 ± 0.30	0.2154	0.7266
2	3	21.59 ± 0.05	0.0370	0.1713
3	3	22.41 ± 0.08	0.0580	0.2589
4	3	15.37 ± 0.05	0.0412	0.2681
5	3	7.97 ± 0.07	0.0500	0.6274

**Table 4:** Integrated analysis of microscopy and PTP2-qPCR for EHP quantification.

Samples	EHP ^#^ Copies/mg	Spore Number * /(field × mg)
1	1.20 × 10^6^	45.23
2	1.08 × 10^6^	39.66
3	6.27 × 10^5^	16.14
4	4.51 × 10^5^	14.78
5	2.36 × 10^5^	13.88
6	1.14 × 10^5^	7.16
7	2.63 × 10^4^	5.91
8	2.22 × 10^4^	3.19
9	1.29 × 10^4^	2.28
10	9.95 × 10^3^	1.56
11	4.34 × 10^3^	0.69
12	8.15 × 10^2^	0.00
13	7.41 × 10^2^	0.00
14	6.84 × 10^1^	0.23
15	2.58 × 10^1^	0.00

**^#^** Conversion formula: copies/mg = (copies/µL) × (50 µL) × (30 mg)^−1^, * The spore number was an average calculated from 20 random fields.
